# Challenges and successes in the sustainment of Dutch community-level smoking cessation interventions for residents with a low socioeconomic position

**DOI:** 10.1186/s12889-023-16529-3

**Published:** 2023-08-23

**Authors:** Nikita L. Poole, Floor A. van den Brand, Marc C. Willemsen, Gera E. Nagelhout

**Affiliations:** 1 Department of Health Promotion, Care and Public Health Research Institute (CAPHRI), Maastricht University, Maastricht, the Netherlands; 2 IVO Research Institute, The Hague, the Netherlands; 3 Department of Family Medicine, Care and Public Health Research Institute (CAPHRI), Maastricht University, Maastricht, the Netherlands; 4 Dutch Expert Centre for Tobacco Control (NET), Trimbos Institute, Utrecht, the Netherlands

**Keywords:** Community, Smoking cessation intervention, Sustainability, Low socioeconomic status

## Abstract

**Background:**

When health promotion interventions are implemented, the gains are often short-lived, as interventions are seldom successfully sustained. The current study explores how and under what conditions community-level smoking cessation interventions for people with a lower socioeconomic position can be sustained, drawing upon interventions delivered in Dutch neighbourhoods with a predominantly low socioeconomic position.

**Methods:**

We conducted 15 semi-structured interviews with key stakeholders from three Dutch community-level smoking cessation interventions implemented at least three years prior. The topic guide was developed based on the Determinants of Innovation framework and transcripts were analysed thematically.

**Results:**

We identified several factors that promote the sustainment of smoking cessation community-level interventions: 1) structural, long-term funding through the commitment of health insurers and policy makers; 2) continued stakeholder enthusiasm and involvement; 3) training and time for professionals to discuss smoking cessation, thereby also increasing the visibility of the intervention for professionals and residents; 4) integrating the intervention with existing initiatives and adapting it to be compatible with current working practices of executive staff; and 5) planning for sustainment as a team from the outset.

**Conclusions:**

The current study highlights challenges and successes in intervention sustainment for people with a lower socioeconomic position. Lack of structural funding was one of the most challenging aspects for intervention sustainment in which health insurers and policy makers can play an important role. Planning for sustainment from the outset would enable intervention coordinators to consider the abovementioned factors early on. This need not be done alone but can best be discussed within a team of stakeholders.

**Supplementary Information:**

The online version contains supplementary material available at 10.1186/s12889-023-16529-3.

## Introduction

Health promotion interventions at the community level are regularly utilised for underserved or disadvantaged groups [1], such as those with a lower socioeconomic position (SEP) and have the potential to improve health equity [2–4]. Population-level improvements in health resulting from community-level interventions often take years to establish, through for instance a gradual change in social norms [5]. Therefore, it is important that effective community-level interventions are sustained over the long-term; however, this sustainment remains a challenge in practice [6].

Sustainability of health promotion interventions is conceptualised by Scheirer and Dearing [7] as the “continued use of program components and activities for the continued achievement of desirable program and population outcomes”. This means, in essence, that interventions need to remain available and accessible to populations in order to result in intervention and population impacts. Several elements have been identified as influential in achieving intervention sustainability, such as the presence of an intervention champion, continued funding and capacity, engaged and supportive stakeholders, perceived benefit by the intervention adopters, and the modifiability and cohesiveness of the intervention with existing systems [8–13]. There is a considerable amount of literature on the sustainability of public health interventions at the community level [10]; however, there is a dearth of research regarding the sustainable recruitment for health promotion interventions and, specifically, the sustainment of smoking cessation community interventions for those with a lower SEP.

Smoking is one of the leading causes of preventable disease and death [14]. Those who smoke regularly often require multiple attempts before they quit successfully [15]. Therefore, it is important that interventions to support cessation have a long-lasting presence in the community where smokers live. Smoking is also more common among people with a lower SEP, such as those with a lower level of education or lower income [16]. There are evidence-based smoking cessation interventions available that match the target group of people with a lower SEP [17, 18] and recruitment strategies that are known to be more successful amongst this group, such as a proactive approach or via word of mouth [19–21]; however, the gains realised from interventions for people with a lower SEP can be short-lived, as interventions are not always successfully sustained in the long-term [6, 9]. As a result, this reduces the (population) impact of the intervention and may also reduce trust and support in the community for new interventions [7, 8]. Furthermore, the initial development and implementation of an intervention often involves considerable start-up costs, which are potentially a poor investment and use of finite resources if the intervention is not sustained [8]. A review of the sustainment of interventions in disadvantaged communities found that only 43% of studies reported interventions that were successfully sustained at least 2 years after the initial training/implementation (defined by the authors as at least half of the original sites/respondents continuing to use the intervention) [6].

Our study focuses on the sustainment of smoking cessation interventions for people with a lower SEP in three urban municipalities in the Netherlands. In the Netherlands, 23.9% of lower-educated adults smoke, compared to 15.3% of higher-educated adults [22]. The three municipalities—Haarlem, The Hague, and Utrecht—have all implemented evidence-based, community-level smoking cessation interventions in a low-income neighbourhood.

By gaining insights from the sustainment of these three community-level smoking cessation interventions, the present study aims to answer the following research question: how and under what conditions are community-level smoking cessation interventions sustained so that they become and remain accessible to residents with a lower SEP? Within this, we also focus on how recruitment of residents with a lower SEP can be sustained.

## Methods

### Design and setting

We conducted semi-structured qualitative interviews (*n =* 15) amongst stakeholders of the three community-level smoking cessation interventions in Haarlem, The Hague, and Utrecht. The three projects were chosen because they were all carried out in districts with neighbourhoods with a predominantly low SEP (based on a combined score of residents’ level of education, labour market participation, and prosperity: standardised disposable income and household wealth [23]). The projects were varied in the type of intervention provided and degree of sustainment. Interviews were performed between March and May of 2022.

In Haarlem, the project ‘Rookvrij Opgroeien’ (Growing Up Smoke-free) was run as a pilot from 2017 to 2019. Alongside the creation of smoke-free areas in the neighbourhood of Haarlem East, individual smoking cessation support was offered in the form of a smoking cessation coach deployed in a general practice. Residents were referred to the cessation coach via their general practitioner (GP or family doctor). At the time of interviewing, the intervention was not active. The project team was exploring funding possibilities to restart the intervention. In The Hague, the smoking cessation group training ‘Voel je vrij!’ (Feel free!), developed by Momentum Training & Coaching, is offered to people who want to quit smoking [24]. Group sessions first covered stress management before moving on to smoking cessation. Participants bore no costs for participating or for using pharmacological support. The project began in 2019 and was still running with temporary funding and funding from the municipality of The Hague at the time of the interviews. In Utrecht, the group training ‘Rookvrij! Ook jij?’ (Smoke-free! You too?), developed by smoking cessation company SineFuma, was given [25] as part of a neighbourhood challenge (‘De Wijkchallenge’) to give up smoking. The project has moved from neighbourhood to neighbourhood, but it is still carried out by the same organisations, funded by the municipality of Utrecht. Participants bore no costs for participating in the interventions or for using pharmacological support. Further details on the approaches in The Hague and Utrecht have been published elsewhere [26].

### Sample

Respondents were recruited via key figures supporting and coordinating the interventions at the three municipalities. Purposive sampling was used to recruit relevant stakeholders in the implementation and sustainment of the three community-level cessation interventions. Most relevant stakeholders were women. Stakeholders included intervention coordinators, implementers, advisors, healthcare professionals, and local municipal officers and were approached via e-mail and telephone (Table 1). All stakeholders were involved in advising, the delivery of, and/or the coordination of the community-level interventions. Of the 25 stakeholders that we approached to interview, ten declined because of time constraints or because they felt insufficiently involved in the recruitment and coordination of the intervention.

**Table 1:** Characteristics of interviewed stakeholders (*n =* 15)

**Characteristic**	**Stakeholders (N)**
**Gender**	
Man	1
Woman	14
**Role**	
Intervention coordinator	4
Intervention implementer	3
Healthcare professional	4
Municipal officer	3
Intervention advisor	1
**Intervention site**	
Haarlem	4
The Hague	6
Utrecht	5

### Data collection

Interviews were conducted by author NP with video conferencing or via telephone and lasted 48 minutes on average (range 28 to 67 minutes). For six interviews, there was a research assistant present to take notes. The audio of all interviews was recorded. Informed consent was obtained from all participants. The interviewing author, NP, was a PhD student with training and experience in conducting qualitative interviews. Prior to the interview, NP had met some of the interviewees during a learning-network meeting for professionals in The Hague, and one interviewee is a colleague of NP, responsible for the research coordination of the intervention based in The Hague. Before the interview took place, participants received the topic list for the interview. At the start of each interview, the research aims were described, as well as the role of the interviewer and the purpose of the interview. All participants agreed to participate in the study and to have the interview recorded and transcribed verbatim. In one interview, two respondents were present. At one respondent’s request, we conducted a second interview to follow up on new developments relevant to our research questions.

We created a topic list based on the Determinants of Innovation (DoI) framework [27] (Supplementary File 1). The framework depicts four processes of innovation (dissemination, adoption, implementation, and sustainment). At any point during the innovation process, progression to the next stage can be affected or influenced by five types of determinants: determinants of the socio-political context, determinants of the organisation, determinants of the adopting person (the user), determinants of the innovation itself, and determinants of the innovation strategy [27]. From this initial work, a list of 29 determinants is presented in the Measurement Instrument for Determinants of Innovation (MIDI) [28, 29]. For our topic list, we made a selection of determinants within the five determinant categories, based on the emerging factors associated with sustainability of interventions at the community level, as identified by Shelton, Rhoades Cooper and Wiltsey Stirman [10]. We paid particular attention to the factor recruitment in our topic list because there is still little known about how recruitment activities can be sustained.

### Analyses

The interviewer and/or the research assistant made notes during the interviews and kept a reflection log to facilitate the analysis. These notes were regularly shared with the rest of the project team. All interviews were transcribed verbatim and returned to the interviewee to be checked and approved without adjustments. Transcripts were subsequently imported into NVivo 12 (QSR International^©^, Melbourne, Australia) for coding and analysis. Transcripts were analysed thematically. A code tree was developed both deductively, based on the DoI model, and inductively by NP. The first five interviews were double coded by NP and the research assistant (*K = 0.83)*. Disparities and disagreements were discussed to reach complete agreement on the coding tree for the first five interviews. The subsequent 10 interviews were divided between NP and the research assistant for final coding, during which any new codes that emerged were shared between the researchers.

The DoI model was used to structure the themes and sub-themes (Table 2). For instance, the theme ‘ determinants of the innovation’ contained sub-themes directly from the model, such as ‘compatibility with current way of working’, and new themes, such as ‘scope of the intervention’. Themes relating to the sustainment of recruitment of participants were included under the themes ‘ determinants of the innovation’, ‘ determinants of the organisation’ and ‘ determinants of the user’. Saturation of all sub-themes was reached, as no new codes were added to the coding tree in the final four interviews coded.

**Table 2:** Determinants of intervention sustainment according to the respondents, presented in the determinants of innovations framework

Level of determinants	Determinants
**Determinants of the socio-political environment**	• Political environment• Health insurance and reimbursement
**Determinants of the organisation**	• Collaboration and engagement of stakeholders • Financial resources • Time available to carry out the intervention • Role of the coordinator • Staff capacity and replacement when staff leave
**Determinants of the user**	• Awareness of content of the innovation • Support from management
**Determinants of the innovation**	• Sustained recruitment • Compatibility of the intervention S• cope and design of the intervention

## Results

Respondents who were part of ongoing interventions were asked about the extent to which the respondents felt their intervention was sustained. They answered that the interventions were not yet fully sustained, which suggests that sustainment itself can be a long and potentially ongoing process.

### Determinants of the socio-political environment

#### Political environment

In 2018, the Dutch government and over 70 societal organisations signed a National Prevention Agreement (NPA), which included policy intentions to address excessive alcohol use, overweight and obesity, and tobacco use. For the theme of tobacco use, the government set a target to achieve a smoke-free society, defined as reaching a smoking prevalence of less than 5%, by 2040. In order to be eligible for governmental grants as part of the Dutch NPA, municipalities can choose two of the three themes (smoking, alcohol, and obesity). A unifying element within the NPA is the Smoke-free Generation campaign, which was launched in 2015 by the Dutch Alliance for a Smoke-free Society (Alliantie Nederland Rookvrij) to stimulate and motivate local communities and professionals to implement smoke-free areas and initiate other activities to protect children from exposure to tobacco and tobacco products [30, 31]. Respondents noted that there is attention and enthusiasm for the smoke-free generation movement amongst municipalities, reflected in the creation of smoke-free playgrounds and other public spaces in an effort to prevent children from starting to smoke but less so for smoking cessation support. This makes it more challenging to keep stakeholders engaged and to secure funding for smoking cessation interventions.

“*I think that municipalities already think it’s very good if they are working towards a smoke-free generation. It gets a lot of attention and [smoking cessation] is just less prominent.” (Coordinator 1)*

One respondent described another aspect: there is a lot of attention for an integral approach—that an individual’s health problems are tackled alongside other underlying or concurrent problems—and smoking is not always seen as necessary or important for policymakers in the municipality to tackle. There is also more enthusiasm amongst health professionals to tackle lifestyle issues together than to focus specifically on smoking. The movement towards an integral approach was seen as positive, but the respondent emphasised that there still needs to be attention for single issues such as smoking.

“*They are still only working on an integrated approach and they say: ‘Yeah smoking cessation, those people have so many problems, they aren’t going to manage it, so we’re going to help them with the other problems first’ but you cannot solve the other problems in one day, so in the end nothing happens for smoking cessation.”* *(Coordinator 1)*

#### Health insurance and reimbursement

A major obstruction to intervention sustainment is the low level of funding available for smoking cessation attempts due to limited health insurance coverage in the Netherlands. As part of the basic insurance package, smokers are entitled to full reimbursement of a behavioural smoking cessation support trajectory with pharmacological support, but only one trajectory per calendar year. Additionally, many patients with chronic conditions are not entitled to this reimbursement as a separate fund is provided to their GP for all of their treatment. This means that often additional and non-structural financial resources are required to allow residents to participate free of charge, otherwise the intervention would remain inaccessible to many smokers with a lower SEP. The rigidity of this regulation was often a frustration for the intervention coordinators.

*“In terms of funding [of smoking cessation support], only once per year and that is of course a fixed amount and you’re really stuck with the existing structures that are there.” (Coordinator 4)*

### Determinants of the organisation

#### Collaboration and engagement of stakeholders

The engagement and collaboration of various parties is required to collaborate on neighbourhood prevention, such as healthcare professionals, neighbourhood professionals, intervention owners, the municipality, and health insurers.

“*The point is that together you all have one goal. [...] You have more strength together, so if you fail for a moment, someone else will take over and carry it on. […] So yes, forming the collaborative group, that is in the making, but that is a condition *[for sustainment]*.” (Healthcare professional 2)*

To ensure that there is continued attention for smoking cessation, respondents suggested that regular meetings with relevant stakeholders in which smoking and cessation are fixed agenda points could help promote sustained attention for the topic. Additionally, intervention champions are key to maintaining enthusiasm amongst stakeholders and breathing life back into an intervention. Intervention champions were identified by the respondents in all three municipalities and had different roles within implementation and coordination of the interventions.

*“At some point the enthusiasm subsides, yeah, then that weakens a bit and then you actually need someone who stimulates that again, who wants to put energy into it again*.” *(Implementer 1)*

#### Financial resources

Across all three municipalities, one of the most challenging aspects of sustaining their intervention was funding. Funding covered participant recruitment, the provision of the intervention, and the wider coordination of stakeholders.

*“The [cessation support] offer is there, everything is there, only it breaks down every time with the funding.” (Healthcare professional 4)*

Respondents described various funding constructions, with much of the funding being temporary in nature. In one municipality, for instance, funding was provided by a national non-governmental funding agency, the municipality, and a health insurer. Adjacent evaluative research activities were also funded by the funding agency. The municipalities were able to financially support the project using grants from the local prevention agreement, but there was uncertainty about how long these grants would be available for; however, project coordinators were in conversation with health insurers to try and secure more structural funding.

*“I’d prefer—although the health insurer is not very open to that—that we create joint financing. A shared savings model, which you invest in together at the front end and you also save together, but that it always flows back into the neighbourhood approach.” (Municipal officer 3)*

#### Time available to carry out the intervention

The temporary nature of funding often had a direct impact on the time available for intervention coordinators to carry out their tasks. When their hours for the intervention ran out, their role in the intervention and, as a result, the intervention itself, came to a standstill.

*“We receive a budget from the municipality to carry out the project, but at some point, the hours run out. I don’t actually have any hours anymore for the intervention.” (Implementer 1)*

In addition, recruiting participants was also named as an especially time-consuming and money-intensive activity, for which resources were sometimes lacking.

As the first point of contact for many residents, GPs can play an important role in recruitment of participants for a community smoking cessation intervention. Both healthcare professionals and intervention coordinators cited a lack of time or interest on the part of the GP to communicate with the coordinators, educate themselves about the intervention, discuss smoking cessation with their patients, and refer them to the intervention.

“*Some GPs are specialists and simply don’t have enough time and some don’t have any interest in delving into it. Yeah, those are shortcomings that prevent you from sustaining such things or helping patients*.” (Healthcare professional 1)

To alleviate the time pressure experienced by GPs, the participating GP practice in one municipality made an agreement with their local pharmacy to handle all cessation medication prescription requests with the health insurers, making intervention referral less time-consuming.

#### Role of the coordinator

The coordinator of the neighbourhood approach is a central point of contact, aiding the flow of information regarding developments in the neighbourhood and maintaining the commitment and collaboration between involved parties. Healthcare professional 1 said:

*“* *It is sometimes very important for a neighborhood approach that you just have a point of contact or someone who can act as a spider in the web and knows what the possibilities are.” (Healthcare professional 1)*

Whilst in two municipalities it was clear who was coordinating the intervention, this was not the case during initial interviews in one of the municipalities. Towards the final interviews, discussions had taken place to determine who would coordinate the intervention going forward. Across the three settings, it was generally agreed that the coordination role can best be done by the municipal health service, a separate entity from the municipality itself focussed specifically on (preventative) health services, rather than the municipality. This is because respondents felt that the municipal health service was better placed to take up the role, with an existing network at the neighbourhood level.

“I think that it is very appropriate [that the municipal health service coordinates it] because smoking cessation in itself is perhaps a treatment, a one-on-one care, which belongs more in healthcare, but it is precisely this collaboration with the neighborhood and the entire network around preventive health at neighborhood level that should arise. I really think that is a role for the municipal health service to contribute to this.” (Coordinator 3)

#### Staff capacity and replacement when staff leave

Respondents also felt that staff capacity plays a role in intervention sustainment.

*“* *I know that the smoking cessation project was first done by one colleague. You can't do that. So now we've done it with the three of us and then it works.” (Implementer 3)*

In some intervention sites, there was a shortage of professionals to carry out the intervention. In addition, one respondent spoke of the lack of priority given to the intervention with many other projects demanding one’s attention.

*“[Healthcare professional] is really searching for collaboration within the neighbourhood, but you notice that they are just short of staff, too few people to carry it out properly. They just really struggle with that and that is still the case.” (Coordinator 1)*

In one instance, staff replacement resulted in more energy being invested into the intervention, but elsewhere, it meant that more time was spent becoming familiar with the intervention and one’s role within it. There were also concerns that the transfer of work to new colleagues risked a loss of knowledge and enthusiasm for the intervention and loss of trust between residents and professionals that have close contact with the neighbourhood.

*“We now have a few nurses who find this theme very important, who are really committed to that and if at some point someone else comes in their place, then you don’t know whether that transfer will go well and whether the new person also finds [the intervention] important.” (Implementer 1)* *“This target group find many changes difficult… some also suffer from trauma, so there are some trust problems: how do I trust someone? Suspicion.” (Implementer 3)*

### Determinants of the user

#### Awareness of the content of the innovation

One important aspect for sustainment when discussing the role of healthcare and neighbourhood professionals is their awareness of the intervention and its contents. Intervention users and implementers noted that there are still many (healthcare) professionals who are not aware of the intervention or how they can use it.

*“Actually, you have to make this offer much more widely known in the neighborhoods, but also to professionals, so that there really is a continuous influx of people who want to quit smoking or who need help or questions about quitting smoking.” (Municipal officer 2)*

As such, the interventions were not yet sufficiently embedded in current practice, and the potential to recruit participants was reduced. In addition, one respondent identified the need among GPs for guidance on which community interventions are available and which ones are covered by health insurance.

*“The awareness of such projects and where you can refer people, that could be so much better. I don't know what is reimbursed, what is not reimbursed.” (Healthcare professional 1)*

#### Support from management

A facilitator of intervention sustainment mentioned by an intervention implementer was the support received from their management. This came in the form of providing enough hours for intervention implementers to carry out the work and being patient when recruitment took longer than expected, where the intervention may otherwise have been discontinued.

*“If management doesn’t support it, then it just doesn’t happen. Management really supported us. Even during [the coronavirus pandemic] it was quite difficult that we had to postpone all the time. They were very understanding of that. And the fact that we ended up reaching fewer people as well, they were understanding of that.” (Implementer 1)*

### Determinants of the innovation

#### Sustained recruitment

Participants struggled to describe how the process of recruitment itself could be better sustained; however, they reported that sufficient and continuous resident participation is important for the intervention’s sustainment.

*“If I stubbornly continue to offer those training courses and only two/three people continue to sign up for it, then that is actually a pity and we can perhaps use [our resources] differently.*”* (Implementer 2)*

Participants spoke about continuous and periodic recruitment procedures. In the site which offered individual training, residents were continuously recruited whereas in the group-cessation sites, recruitment was done periodically. This meant that the energy and attention for the intervention and smoking cessation had to be reignited for a new round of recruitment.

*“I think that's also the big challenge of the project, because yes, it's a bit campaign-like [...] at some point those courses are over, then the recruitment for those courses also stops and what you really want is that at that moment the professionals are so aware of the theme, that they also continue.” (Implementer 1)*

Respondents from Haarlem did not report any difficulties with recruitment.

Two factors mentioned that aid recruitment were the visibility of the intervention and that residents trust the professionals approaching them about smoking cessation and the intervention. In The Hague, respondents reported that the visibility of the intervention—what it entails and what it aims to deliver—was lacking among residents. Without improving visibility, the intervention was at risk of only reaching residents who already have closer contact with the municipality and social services.

*“People just don’t know that [the intervention] exists. It isn’t visible.” (Coordinator 2)*

According to one respondent, a way to increase visibility is to link the intervention to a GP practice in the neighbourhood as much as possible “*because then it is recognisable to the patient*” (Healthcare professional 4). In two municipalities, neighbourhood health ambassadors were also trained to discuss smoking with residents to increase intervention visibility and opportunities to recruit. This was because neighbourhood health ambassadors lack self-efficacy in discussing smoking cessation. They were reportedly also concerned about the damage discussing smoking cessation would have on their trusting relationship with residents.

*“People from the neighbourhood team, for example, a sports district worker or a welfare coach […] find it difficult to get started with this topic. But it would be great if they could function as a referrer, or at least a signaller, and they could point people to the intervention that is offered. And that's what we're now investing in to actually improve that signalling even more, because not everyone comes to the doctor, of course*.*” (Municipal officer 3)*

Respondents also recognised that being approached by someone who is known and trusted increases the likelihood that a resident will participate. This effect is strengthened if approached by multiple trusted people, and in the case of people with a lower SEP, this is often family, neighbours, and friends. They elaborated that this is because trust in the government and health agencies is at times lacking amongst people with a lower SEP.

“*Initially, there is a lot of resistance and eventually, yeah, gaining some kind of trust, I think is very important [...] So it also takes a lot of time to gain that trust from the neighborhood.” (Municipal officer 3)*

In practice, recruitment strategies entailed the involvement of key members of the community and trusted healthcare professionals, along with the utilisation of various trusted community settings, to promote the intervention.

*“* *I have also contacted the Salvation Army, the neighborhood team, to tell them what the possibilities are, for example, if there would be another smoking cessation intervention, because then you can start recruiting people again.” (Healthcare professional 3)*

#### Compatibility of the intervention

Another determinant of sustainment is the extent to which the intervention is compatible with what is currently offered and the current ways of working. In one municipality, respondents spoke about the many public health interventions present and how their intervention needs to be well-established and integrated within the existing initiatives.

*“Of course, you also want to connect with what is already happening and that is now what is finally happening [for our intervention].” (Coordinator 1)*

Another respondent expressed their exasperation at trying to encourage municipal workers to incorporate conversations about smoking into their interactions with residents.

*“* *I think I asked a number of people from the municipality ten times: 'You speak to these people regularly, wouldn't you...' 'No, no, no, difficult, difficult, difficult, difficult.' Yes, everything also works in time blocks so I can't blame them either. It really requires a different way of working.” (Municipal officer 1)*

Another aspect of compatibility is the extent to which the intervention fits the perceived needs of the users. In one municipality, adoption of the group intervention amongst healthcare professionals was low as they focussed instead on promoting the individual cessation support that they already offered. This made it more difficult to integrate the intervention into standard practice.

*“It was an intervention from the outside, which does not directly match the demand from those professionals in the neighbourhood. […] Such a group offer fits best in a practice that doesn’t do anything themselves, but not in a practice that is already very active. And this was a practice that was very active, so to that practice it was kind of a threat to their own offerings.” (Municipal officer 2)*

#### Scope and design of the intervention

Another aspect of the intervention itself that could influence its sustainment is the scope of the intervention. In one municipality, some respondents recognised that the area they had chosen for the intervention was too large for a community-level intervention in that there were insufficient resources (namely time and staff) to maintain the intervention at that scale. In addition, it was believed that the use of a group training made recruitment more challenging. One participant suspected that this could be because the residents were not ready to participate in a group.

*“Our experience with another group that we tried in a group was that the recruitment for this is quite difficult.* *People find the step is actually too big* *.* *” (Healthcare professional 4)*

Lastly, a respondent involved in the implementation of the intervention admitted that they had not sufficiently considered how the intervention itself could be sustained.

*“* *If this works, how can we keep doing it? That's actually the step I skipped, which is bothering me now.” (Municipal officer 2) *

The intervention was not implemented with the aim to sustain it, but the respondent felt that both they and the stakeholders they collaborated with could have been more attentive to this point.

“*Sometimes setting up such a project requires so much attention that you forget to think about: oh yes, it also has to be sustained.” (Municipal officer 2)*

## Discussion

This study identified important determinants that influence the sustainability of community-level smoking cessation interventions. At the socio-political level, the main determinants relate to the rigid and limited funding for smoking cessation by the health insurance system and the temporal and limited funding from the municipality and the national government for carrying out these interventions. Furthermore, political attention for smoking cessation is important for securing long-term funding and the engagement of stakeholders. At the organisational level, there were major concerns around the provision of sufficient resources to sustain the interventions. Awareness of the interventions and their contents and receiving support from management were main determinants for sustainment at the user level. Lastly, respondents highlighted key areas in which the intervention itself may influence its sustainability, such as the degree to which it is compatible with what is currently offered and the current working practices.

The role of many of the determinants identified by the respondents were already found by previous studies of (public) health intervention sustainability, not specific to smoking cessation [6, 9, 11, 13]. Several of these factors, such as the need for adequate resources (time, staff, funding) have also been identified as important for the implementation and long-term enforcement of tobacco control legislation at the local level [32]. In scarcity of such resources, tobacco control interventions may become de-prioritised in favour of other issues, negatively impacting their sustainment [33]. From the interviews in the present study, it was clear that funding was one of the most challenging aspects for intervention sustainment and that this influenced several other determinants at the organisational level; however, it is important to note that the provision of long-term funding on its own is not sufficient for intervention sustainment. An engaged and committed team, sufficient capacity, and clear coordination are also needed.

Staff capacity and turnover are other major determinants in sustainment. Respondents perceived staff capacity to be affected by various factors, including temporary funding, limiting the number of budgeted hours received to carry out intervention-related tasks, chronic staff shortages, and high workloads experienced by many healthcare professionals [34, 35]. Staff turnover is a risk to sustainability inherent in any community-level intervention; however, a lack of qualified staff may be a factor more prevalent in disadvantaged neighbourhoods [6, 36], where the availability and ability to attract and retain staff can be affected by restricted funding [36].

The study highlighted how some determinants can play a greater role than others in the success of intervention sustainment and that this can depend on the surrounding context. It is therefore important to conduct an assessment of the local context to identify the facilitators and threats to sustainment unique to that context. Local contexts are dynamic [37] and so repetition of this assessment may be necessary.

This study also explored the determinants that influence sustained recruitment for a smoking cessation intervention, which is a lesser studied area. Respondents were able to give many examples and strategies of how to reach smokers with a lower SEP. Respondents also highlighted the importance of a trusting relationship between residents and researchers/implementers [26, 38, 39]. Despite this, in two of the three sites, difficulty remained in recruiting enough participants. In the two sites, group-based interventions were used which were characterised by periodic, rather than continuous recruitment, and commencement at pre-determined dates. Motivation to participate may abate in the waiting time between initial recruitment and the start of the intervention. A low level of enrolment may also suggest that a group-based intervention does not (entirely) match the needs of the target group or that recruitment activities were not sufficient.

Moreover, few insights were given into how the recruitment of residents could actually be sustained; however, participants did report that a periodic recruitment procedure was intensive and required proactively reviving attention and enthusiasm for the intervention. Participants did not explicitly identify this as impacting the sustainability of recruitment; however, from these findings, we hypothesise that the use of less-intensive recruitment methods, such as a continuous recruitment procedure, may strengthen the recruitment sustainability. This hypothesis should be investigated further, with attention for the recruitment of disadvantaged groups for whom participation is typically lower and recruitment strategies are more intensive [21, 40].

As mentioned, de-prioritisation of smoking cessation interventions in favour of other issues is a risk to sustainment, particularly in settings where limited resources must be shared. One could argue that this is an even greater risk in low SEP communities, where the prevalence of other problems such as chronic stress may be higher and thus competing for attention. In the Netherlands, we see that smoking cessation in low SEP communities is newly prioritised, with governmental funding to support 45 neighbourhoods across the country in a programme to develop, implement and sustain smoking cessation interventions [41]. A central component to the programme is its integral approach, which addresses other problems such as stress or financial insecurity in addition to smoking cessation. This is one way of stimulating smoking cessation but not at the expense of other issues, and vice versa.

Whilst the prioritisation of smoking cessation is a promising development, it is important to note that this may be unique to the Netherlands, where the Smoke Free Generation movement has broad political and public support, supported by an active and engaged civil society [42]. As such, the broader societal context in other European countries may play a different role in the sustainment of such community interventions than is presented here in the Dutch context.

## Implications

From the findings, we make the following recommendations for improved sustainment of current and future community-level smoking cessation interventions: 1) secure structural, long-term funding for interventions through the political and financial commitment of health insurers and policy makers—in doing so ensuring that there is sufficient capacity to carry out the intervention as needed; 2) maintain enthusiasm and involvement amongst stakeholders by meeting regularly and providing updates on progress and success stories, as intervention champions can foster stakeholder engagement with the intervention; 3) supporting (healthcare or neighbourhood) professionals in their role by offering training and time to discuss smoking cessation, thereby also increasing the visibility of the intervention for professionals and residents; 4) ensure the intervention is adapted so that it is integrated with existing initiatives and compatible with the current working practices of executive staff (done for example by performing a needs assessment among the main stakeholders or by ensuring that stakeholders are continually involved in the development and implementation of the intervention in a co-creation process); and 5) planning for sustainment from the outset would enable implementers to consider these aspects early on. Implementers do not need to do this alone but can discuss these matters within their team of stakeholders.

## Limitations

A possible limitation of our study is that the community-level smoking cessation interventions at each of the three sites were not fully sustained according to the respondents at the time of interviewing. On the one hand, this could mean that respondents may have had limited insight into the challenges and facilitators to sustainment in the longer-term; however, respondents were able to recognise the challenges faced in the transition towards intervention sustainment. In addition, the study only includes three sites, all in urban settings. The inclusion of additional and non-urban settings would have enabled deeper analysis of the context and comparison of intervention types and their impact on sustainment. Another limitation is that some of the findings may not be generalisable to other countries, particularly with respect to funding sources available at the community-level, the role of health insurers in smoking cessation, and the role of the municipal health service; however, many of our findings are supported by international literature on intervention sustainment [8–13]. Lastly, not all transcripts were double coded.

## Conclusions

The current study highlights challenges and successes in the sustainment of community-level smoking cessation interventions. Successes included the use of intervention champions to foster stakeholder engagement, receiving support from management to continue the intervention despite initial difficulties, and arrangements made to reduce the workload burden on GPs. Major challenges to intervention sustainment were the temporary nature and lack of structural funding, followed by a lack of engagement by stakeholders. The issue of funding is both local to the intervention and system-wide, impacting other aspects of sustainment, such as staff capacity and time available for recruitment and coordination tasks. Where intervention coordinators currently use various temporary funding constructions, health insurers and policy makers could step in to provide opportunities for structural funding; This could start with, but should certainly not be limited to a permanent capacity for intervention coordination, participant recruitment, and the reimbursement of more than one supported smoking cessation attempt per year.

### Supplementary Information

**Additional file 1.**

## Data Availability

The data that support the findings of this study are available on request from the corresponding author NP. The data are not publicly available due to them containing information that could compromise research participant privacy.

## Abbreviations

SEP: Socioeconomic position
GP: General practitioner
DoI: Determinants of Innovation
MIDI: Measurement Instrument for Determinants of Innovation
NPA: National Prevention Agreement
