# Supplementary material for: Challenges and successes in the sustainment of Dutch community-level smoking cessation interventions for residents with a low socioeconomic position
Source: BMC Public Health. 2023 Aug 23;23:1605. doi: 10.1186/s12889-023-16529-3 (PMC10464105; doi:10.1186/s12889-023-16529-3)
Supplement: Supplementary file 1 — Additional file 1. [file 12889_2023_16529_MOESM1_ESM.docx]

| **Section** | **Questions** |
| --- | --- |
| **Opening questions** | 1. Could you first briefly explain what your role is in the (implementation of the) neighbourhood approach and since when you have been involved? 2. Can you first tell us a bit about smoking the neighbourhood approach at the moment? Which evidence-based interventions are used in your organization/municipality? How is that paid for and organised at the moment? 3. Which elements of the apporach are still being carried out today? Why? 4. What made it easier to sustain the elements from the neighbourhood approach? To what extent do you believe that sustaining the neighbourhood approach has been successful? 5. What made it more difficult to sustain the neighbourhood approach? |
| **Recruitment** |  |
|  | 1. Can you briefly explain the recruitment strategy of your organisation or in your neighbourhood/municipality? To what extent (and in what way) is it currently aimed at people with a lower socioeconomic position? 2. Have you seen any developments in recruitment? Why did they come about? 3. What steps have been taken to continue and sustain the active recruitment of people with a lower socioeconomic position? How can this be improved? / What would be needed to continue doing this over a longer period of time as an organization/district/municipality? 4. What has ensured that it was possible to sustain the (intensive) recruitment of people with a lower socioeconomic position? 5. What made it difficult to sustain the (intensive) recruitment of people with a lower socioeconomic position? 6. How can we stimulate the provision of this cessation offer by professionals?   You have already mentioned what made it easier or more difficult to sustain the smoking cessation offer and recruitment. We want to go through a number of different levels and topics to see if we have missed anything. |
| **Determinants** | ***Ask when applicable…*** |
| *Characteristics of the socio-political context* |  |
| Legislation and regulations | 1. To what extent does the neighbourhood approach aimed at smoking cessation fit within existing laws and regulations/policy intentions/regulations in your municipality? 2. To what extent does the neighbourhood approach to smoking cessation, intensive recruitment and attention for people with a lower socioeconomic position fit within the political climate of your municipality? Have there been any changes in recent years? |
| *Characteristics of the organisation* | What are characteristics of the organisation of the smoking cessation intervention that are important for sustainment? |
| Staff capacity and replacement when staff leave | 1. How do staff capacity and staff turnover affect the maintenance of the neighbourhood approach? |
| Coordinator | 1. Who coordinates the neighbourhood approach to smoking cessation in your organisation and who does this in the entire neighbourhood/municipality? 2. What do you think the coordinator of the neighbourhood approach should pay attention to when it comes to sustaining the neighbourhood approach? |
| Resources – financial, time, material, facilities | 1. Do you have sufficient resources at your disposal to sustain this approach and recruitment? (think of financial, time, material and facilities) |
| *Characteristics of the user* |  |
| Sociale steun | 1. Do you think that the management of your organization gives sufficient support to those who carry out the neighbourhood approach and recruitment? To what extent does management support play a role in sustaining this neighbourhood approach? |
| Kennis | 1. Do you think there is enough expertise among those who carry out the neighbourhood approach and recruitment to sustain it? Is this expertise easily transferable? |
| *Characteristics of the innovation* |  |
| Complexity | 1. Do you think the neighbourhood approach is simple enough to continue offering it in the long term? / Would the neighbourhood approach be easily transferable to new stakeholders/municipalities? |
| Compatibility | 1. To what extent does the neighbourhood approach fit well with the current working method of the municipality (or your organisation) and with the available range of smoking cessation support? |
| Relevance for client | 1. Do you think the current neighbourhood approach is relevant and fits well with the users? If not, why? 2. Do you think the current neighbourhood approach is effective for residents with a lower socioeconomic position? 3. Do you expect certain developments in the type of users? |
| **Closing questions** | 1. What recommendations would you make if you had to advise another municipality on implementing and sustaining a neighbourhood approach for smoking cessation? With multiple recommendations: What is the most important thing that you would advise them? 2. Is there anything else you'd like to add? |
